# Feed-forward inhibition: a novel cellular mechanism for the analgesic effect of substance P

**DOI:** 10.1186/1744-8069-1-34

**Published:** 2005-11-18

**Authors:** Long-Jun Wu, Hui Xu, Shanelle W Ko, Megumu Yoshimura, Min Zhuo

**Affiliations:** 1 Department of Physiology, Faculty of Medicine, University of Toronto, University of Toronto Centre for the Study of Pain, Medical Science Building, 1 King's College Circle, Toronto, Ontario M5S 1A8, Canada

## Abstract

Substance P (SP) is a neuropeptide well known for its contribution to pain transmission in the spinal cord, however, less is known about the possible modulatory effects of SP. A new study by Gu and colleagues, published in *Molecular Pain *(2005, 1:20), describes its potential role in feed-forward inhibition in lamina V of the dorsal horn of the spinal cord. This inhibition seems to function through a direct excitation of GABAergic interneurons by substance P released from primary afferent fibers and has a distinct temporal phase of action from the well-described glutamate-dependent feed-forward inhibition. It is believed that through this inhibition, substance P can balance nociceptive output from the spinal cord.

## 

The spinal cord dorsal horn is one of the relay stations for nociceptive information transmitted by peripheral sensory afferents. Some of these sensory afferents are substance P (SP) and glutamate-containing A_δ_- and C- fibers. Upon noxious stimulation, particularly intense stimulation, tachykinins such as SP and neurokinin A (NKA) are released from primary afferent fibers and excite dorsal horn neurons via activation of the neurokinin-1 and neurokinin-2 receptors (NK1R and NK2R), respectively [1,2]. A series of studies have established a role for SP in the transmission of pain information [3,4]. Mice genetically engineered not to express the precursor of SP [4] and mice that do not express SP's target, the NK1R [3], both display reduced responses to painful stimuli. Despite these promising initial findings, NK1R antagonists have failed to produce analgesia in a variety of clinical pain models [5]. One possible explanation for these inconsistent results is that SP may produce mixed effects in sensory-related transmission and modulation. Indeed, Mohrland and Gebhart reported that an intrathecal injection of SP had antinociceptive effects [6]. Similarly, a study has found an analgesic effect mediated by SP and further suggested that it might be mediated by μ-2 opioid receptors [7]. Other studies demonstrate an interaction between tachykinin and opioid systems, lending support for a role of opioid receptors in SP-mediated antinociception [8].

Although many studies have highlighted the importance of SP in pain transmission, the synaptic mechanisms underlying the antinociceptive effect of SP remain unclear. In a recent study published in *Molecular Pain*, Gu and colleagues used a combination of electrophysiological, pharmacological, genetic and behavioral techniques in rats and found that SP can modulate inhibitory transmission in lamina V of the spinal cord dorsal horn, thereby exerting an analgesic effect on nociceptive sensory processing [9]. Through this finding, the study provides novel insight into the role of SP in the spinal cord dorsal horn, which has important implications for the therapeutic control of pathological pain.

## SP drives glutamate-independent feed-forward inhibition

The neuronal network in the spinal dorsal horn is extremely complex. Within this circuit, inhibitory interneurons containing GABA and/or glycine play important roles in controlling network excitability. Activation of these neurons can initiate feedback inhibition and feed-forward inhibition, which are believed to be critical for the fine tuning of sensory information at the spinal level [10]. SP and glutamate, present in some primary afferent fibers that respond to painful stimuli, can mediate excitatory responses in postsynaptic dorsal horn neurons [1,11]. Although it had been shown that glutamate can bind to receptors on GABA and glycine-releasing interneurons to decrease nociceptive transmission, the possibility that SP could also drive inhibitory activity in the spinal cord dorsal horn had not been explored.

Gu and colleagues stimulated the dorsal root of the spinal cord, which contains primary afferent fibers, with high frequency stimulation to cause the release of both SP and glutamate in the presence of N-methyl-D-aspartate (NMDA) receptors and non-NMDA receptors antagonists. They found a robust and long-lasting increase in both the frequency and amplitude of spontaneous inhibitory postsynaptic currents (sIPSCs). Similar results were obtained with chemical stimulation using capsaicin, which also excites primary afferent fibers to release both glutamate and SP [9]. This increase in sIPSCs when glutamate-mediated excitatory responses were blocked suggests that glutamate-independent feed-forward inhibition exists in the dorsal horn.

Could SP be responsible for glutamate-independent feed-forward inhibition? First, among different neuropeptides such as galanin, neuropeptide Y, somatostatin, calcitonin gene-related peptide and SP tested, only SP enhanced the frequency and amplitude of sIPSCs. These results demonstrate that the effect of SP is selective. Second, application of NK1R antagonists blocked both electrical and chemical stimulation-induced increases of sIPSCs. Third, exogenously applied SP- or stimulation-induced increases of sIPSCs were abolished in NK1R knockout (NK1R^-/-^) mice. Taken together, these results demonstrate that increases of SP can drive inhibitory activity in the dorsal horn of spinal cord.

To further explore the neuronal mechanisms underlying SP-driven feed-forward inhibition, pharmacological tools were used to show that pertussis toxin-sensitive G proteins such as Gi and Go are involved in SP-driven feed-forward inhibition. Moreover, GIN mice, a strain of transgenic mice that express enhanced green fluorescent protein in GABAergic neurons, were used to show that SP can directly excite some GABAergic neurons by inducing prolonged depolarization, firing of action potentials, and increases of intracellular Ca^2+^. Since SP had no effect on both miniature inhibitory postsynaptic currents (mIPSC) and evoked inhibitory postsynaptic currents (eIPSC), SP-driven feed-forward inhibition is more likely due to the direct excitation of GABAergic interneurons by SP releasing primary afferent fibers than to action potential-independent modulation of activity within the synapse.

**Figure 1:**
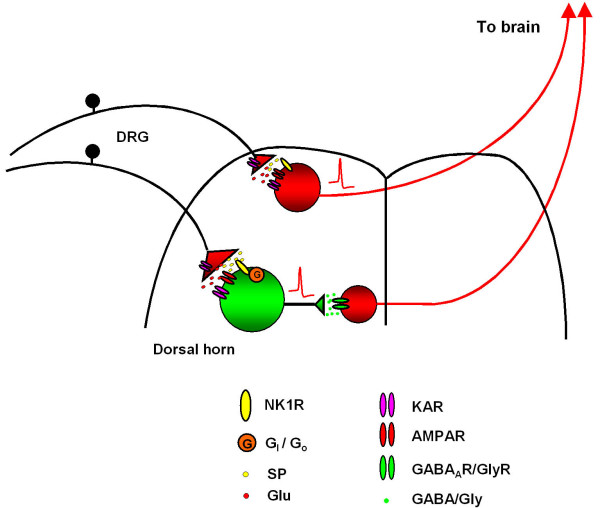
**Schematic diagram of SP-driven feed-forward inhibition in lamina V of the spinal cord dorsal horn**. Sensory information starts from dorsal root ganglion (DRG) neurons, is relayed by spinal cord dorsal horn neurons and then is projecting to the brain. The intense painful stimulation of primary afferent, mostly A_δ_- and C- fibers, induced the release of SP in lamina I and V. On the one hand, SP directly excites projection neurons in laminar I, thereby inducing pronociceptive response. On the other hand, SP in laminar V excites inhibitory interneurons in lamina V, through NK1 receptor (NK1R) and the following signaling pathway involved pertussis toxin-sensitive G_i_/G_o _protein and possible downstream targets Ca^2+ ^or K^+ ^channels. The firing of these interneurons releases GABA and/or glycine, activate GABA_A _receptor (GABA_A_R) and/or glycine receptor (GlyR), and initiates feed-forward inhibition in the projection neurons ascending to the brain. The inhibitory interneuron is in green and the projection neuron is in red.

SP-driven feed-forward inhibition is summarized in Figure 1. The intense painful stimulation of primary afferents, mostly A_δ_- and C- fibers, induces the release of both glutamate and SP, mainly in lamina I and V. In lamina I, SP release activates projection neurons that relay pain-related information to higher centers in the nervous system to process the different qualities of the stimulus. In lamina V, SP binds pertussis toxin-sensitive G_i_/G_o_-coupled NK1R on inhibitory interneurons, which leads to a cascade of downstream events that may result in changes in the activity of non-selective cation channels or inwardly rectifying potassium channels [12] ultimately leading to GABA and/or glycine release. This could initiate feed-forward inhibition in the projection neurons and probably feed-back inhibition on the primary afferent fibers.

## Different temporal phases between SP- and glutamate-driven feed-forward inhibition

Even though SP coexists with glutamate in primary nociceptive afferent synaptic terminals, their roles in various pain conditions may be distinct considering that higher frequencies of primary afferent stimulation are required to evoke the release of SP compared to glutamate [13]. Consistent with this idea, previous electrophysiological studies reported that activation of these fibers produced two phases of excitatory postsynaptic potentials (EPSPs), a fast EPSP mediated by non-NMDA receptors and a slow EPSP possibly mediated by the NK1R and NK2R [1,14]. Therefore, it is conceivable that SP-driven feed-forward inhibition and glutamate-driven feed-forward inhibition have different temporal phases.

To address this issue, Gu and colleagues first studied SP-driven feed-forward inhibition in the absence of glutamate receptor antagonists [9]. Their results showed that high frequency stimulation induced a robust and long-lasting increase in sIPSCs in wild-type mice but not in NK1R^-/- ^mice. However, electrical stimulation-induced immediate eIPSCs, which could be blocked by glutamate receptor antagonists and are believed to be glutamate-driven feed-forward inhibition, were similar between wild-type and NK1R^-/- ^mice. Taken together, these results provide evidence for a long-lasting, glutamate-independent SP-driven feed-forward inhibition that is distinct from the immediate pulse-by-pulse glutamate-driven feed-forward inhibition.

## Functional significance of SP-driven feed-forward inhibition

After confirming the existence of the SP dependent feed-forward inhibition as well as delineating its distinct temporal phase, Gu and colleagues searched for the functional significance of the SP-driven feed-forward inhibition in the spinal cord dorsal horn. They hypothesized that the SP-driven feed-forward inhibition may serve to balance neuronal activity by counteracting SP-mediated excitatory nociceptive responses. This hypothesis challenges the traditional role of SP as a purely "pro-pain" substance in favor of a more homeostatic role.

A clever set of experiments were devised to address this question. First, SP-mediated nociceptive transmission in the superficial dorsal horn was blocked with a selective lesion of NK1R-expressing neurons using an intrathecal injection of SP-conjugated saporin (SP-SAP) [17,18]. It is important to note that the SP-SAP injection does not cause cytotoxicity in lamina V neurons so the SP-driven feed-forward inhibition remained intact. If an antinociceptive role for SP-driven feed-forward inhibition exists, the activation of SP release would have an analgesic effect in SP-SAP injected animals but blocking SP transmission would result in behavioral sensitization in SP-SAP injected animals.

Behavior responses to nociceptive heat stimuli were studied in SP-SAP treated animals after capsaicin treatment and intrathecal application of NK1R antagonists, resulting in two major findings [9]. First, capsaicin-induced behavioral sensitization in control animals was attenuated in SP-SAP treated animals consistent with the role of SP transmission in the superficial laminae as being "pro-pain". Second, NK1R antagonists reduced behavioral sensitization in the control group whereas the opposite effect was found in SP-SAP treated rats that support a possible antinociceptive role for SP in lamina V, probably through feed-forward inhibition. These results may explain hyperstimulation-induced analgesia, in which pain is controlled through additional painful stimulation or through counter irritation [9,19], and the lack of an analgesic effect of NK1R antagonists administered in clinical trials [5]. This study portrays SP as a double-edged sword for pain transmission, SP's actions may depend both on the location of release (lamina I *vs *lamina V) and an the type of neuron it synapses with (projection neurons *vs *inhibitory neurons).

## Future directions

The elegant study by Gu and colleagues proposes a novel cellular mechanism for the neurokinin system in pain transmission and modulation. Dissecting its intricacies will not only contribute to a better understanding of how somatosensory inputs, including pain information, are coded within the spinal dorsal horn, but may also foster the development of more efficacious treatments for pain control.

Due to the important implications of SP-driven feed-forward inhibition in nociceptive transmission, some limitations of these findings need to be addressed. (1) SP can be released from at least three different sources: primary afferent fibers, descending projections fibers and local neurons. The current study uses dorsal root stimulation so it is likely that the primary afferents are the cause of the SP release [20,21]. Since it is possible that SP has a different function depending on its source, this study cannot rule out the other potential actions of SP when it is released from local neurons or descending terminals. (2) Activation of the NK1R in lamina V induced interneuronal depolarization and firing that was not due to action potential-independent synaptic transmission. Therefore, these receptors are unlikely to be localized in the presynaptic terminal. It would be interesting to investigate two related issues. One is the exact location of NK1Rs on the neuron, since some studies report that NK1Rs are postsynaptically or extrasynaptically located [22]. The other issue is the molecular mechanism for SP induction of interneuronal excitability. It is thought that SP might directly induce a postsynaptic current or indirectly modulate various receptors and channels. The G_i_/G_o_-coupled NK1R is reported to activate phospholipase A2 and mobilize arachidonic acid [23]. The final targets for the signaling pathway initiated by SP binding to NK1R to result in SP-driven feed-forward inhibition in the spinal cord dorsal horn are still a mystery. (3) Although SP and NKA are synthesized together, they affect spinal nociception in different ways [24, 25]. Still, it cannot be ruled out that NKA might play a role in SP-driven feed-forward inhibition. The study excludes the possible involvement of neurokinin B (NKB) in SP-driven feed-forward inhibition since the NK3R (the receptor for NKB) antagonist did not significantly attenuate capsaicin-induced increases of sIPSCs. (4) This study showed that a long lasting increase in sIPSCs is SP-dependent but glutamate-independent. This conclusion is based on pharmacological studies using ionotropic glutamate receptor antagonists. However, whether metabotropic glutamate receptors are involved in SP-driven inhibition remains unknown. The authors cannot prove without a doubt that SP-driven feed-forward inhibition is glutamate independent without first excluding the potential role of metabotropic glutamate receptors.

Although the complexity of nociceptive transmission and modulation in the spinal cord make it difficult to address all questions in a single model, we believe the description of a SP-driven feed-forward inhibition to be a crucial finding towards the understanding of the role of SP in sensory transmission, modulation and plasticity.
